# An Analysis of the Operation Factors of Three PSO-GA-ED Meta-Heuristic Search Methods for Solving a Single-Objective Optimization Problem

**DOI:** 10.1155/2022/2748215

**Published:** 2022-10-14

**Authors:** Ali Fozooni, Osman Kamari, Mostafa Pourtalebiyan, Masoud Gorgich, Mohammad Khalilzadeh, Amin Valizadeh

**Affiliations:** ^1^Foster School of Business, University of Washington, Seattle, WA 98105, USA; ^2^Department of Business Management, University of Human Development, Sulaymaniyah, Iraq; ^3^Department of Industrial Engineering, University of Science and Culture, Tehran, Iran; ^4^Department of Industrial Engineering, Velayat University, Iranshahr, Iran; ^5^CENTRUM Católica Graduate Business School, Lima, Peru; ^6^Pontificia Universidad Católica del Perú, Lima, Peru; ^7^Department of Mechanical Engineering, Ferdowsi University of Mashhad, Mashhad, Iran

## Abstract

In this study, we evaluate several nongradient (evolutionary) search strategies for minimizing mathematical function expressions. We developed and tested the genetic algorithms, particle swarm optimization, and differential evolution in order to assess their general efficacy in optimization of mathematical equations. A comparison is then made between the results and the efficiency, which is determined by the number of iterations, the observed accuracy, and the overall run time. Additionally, the optimization employs 12 functions from Easom, Holder table, Michalewicz, Ackley, Rastrigin, Rosen, Rosen Brock, Shubert, Sphere, Schaffer, Himmelblau's, and Spring Force Vanderplaats. Furthermore, the crossover rate, mutation rate, and scaling factor are evaluated to determine the effectiveness of the following algorithms. According to the results of the comparison of optimization algorithms, the DE algorithm has the lowest time complexity of the others. Furthermore, GA demonstrated the greatest degree of temporal complexity. As a result, using the PSO method produces different results when repeating the same algorithm with low reliability in terms of locating the optimal location.

## 1. Introduction

A nongradient optimization method is a stochastic method, which means that, unlike gradient optimization, the results are heavily randomized. A scenario similar to Darwinian evolution is simulated in which the closest point to a maximum or a minimum value is selected as the optimal point in a function [1–4]. Unlike gradient methods, evolutionary optimization does not heavily rely on mathematics, and the initial starting point does not have nearly as much impact. Because of the random nature of evolutionary optimization, it is mostly less efficient than gradient-based optimization since it does not even guarantee an optimal solution [5, 6]. However, the method is more aggressive and considers more solutions than gradient methods do, allowing it to find multiple local minima points, which give it some advantages. The way evolutionary optimization works is that first, one must generate a mathematical function to create a scenario with specific conditions and then various points will be randomly plotted throughout the function in ideal locations [7–10]. The results will be compared and then used to converge throughout the function. These results then adapt and converge toward the optimized points chaotically through trial and error. The step size for updating unknowns is generally required when applying gradient-based optimization algorithms [11, 12]. To achieve better generalization and convergence, learning rate scheduling schemes have been used in addition to the fixed learning rate. Staircases [13] and exponential decay [40] are simple, but popular schemes for reducing stochastic noises. AdaGrad [14], AdaDelta [15, 16], RMSprop [17], and Adam [18] have also been developed for parameterwise adaptive learning rate scheduling. So while finding the optimal point is not guaranteed, it is at least possible to find these points' potential locations.

Since evolutionary optimization has a variety of starting points, it is not subject to the same weakness as gradient optimization. Gradient optimization accurately converges on the local minima. The function, however, does not know whether it has reached the global minima. As a result, less-optimal solutions are often reached than what is possible [18]. With evolutionary optimization, starting points are all across the function, which raises the probability of one starting near the global minima. They all converge toward their local minima, and the results are then compared. Based on these, we can more easily approximate the global minima within the bounds of our function. The best results, in general, can come from combining gradient and nongradient-based optimization to converge on the best solution, for this one would start with the broad function and implement evolutionary optimization [19, 20].Despite the fact that it is not very analytical, it would often instinctively converge near the global minima, providing an indication of the general location. Afterward, a gradient-based algorithm may be used with the determined area as a starting point. Using a mathematical function, it will converge toward the global minima and provide an accurate result. It is possible to find the global minima for any function by combining the two algorithm types accurately (see Table 1).

Multiobjective optimization has been applied in many fields of science, including engineering, economics, and logistics where optimal decisions need to be taken in the presence of trade-offs between two or more conflicting objectives. There are many applications in computer science such as cloud computing [28–30], image processing, [31], medical science [32], robotics and mechanics [33], controller design [34], wireless sensor network [35–37], architectural design [38], and metaheuristic methods convergence [39, 40]. There are some other applications for prediction methods, Feynman's Path Integral [41], Semantic Segmentation [42], Internet of things [43, 44], Signal processing [45], distributed networks [46, 47], and Software Defect Prediction [48]. Some other optimization methods are adaptive regeneration framework [49], Mean Extra-Gradient [50], Bi-LSTMC [51], random key genetic algorithm [52], and Complementary-Label Source Domain [53]. Moreover, signal processing fields include Ultrawideband Rejection [53], GaAs technology [54], Visual question answering [55], Visual Reasoning [56, 57], Semantic Network [58, 59], attack detection [60], Smart Homes [61], Fog computing [62], Neural Tracking [63], light detection [64], Buffering Algorithm [65], decision making [66], classification [57, 67–69], Growth Cycles [70], Remote sensing [71], power generation [72–74], vehicle routing problem [75], and structure design [76, 77].

## 2. Methods and Materials

### 2.1. Genetic Algorithm

The genetic algorithm is a learning program that mimics natural evolution concepts such as reproduction crossover and mutation to produce what the program considers optimal offspring. It is the most general type of evolutionary optimization. It takes the general ideas behind it and puts them into action. It starts with various points spread randomly throughout the function, taking into account the various possible solutions within the problem's parameters. It allows the program to consider various possible solutions and focus on each of them to determine the best one. Once the algorithm has its values, it calculates each solution's fitness generated in the function. Then a pair of solutions can be selected so long as they increase the chances of generating offspring; each parent can be used more than once per iteration to generate offspring. Once the points are selected, cross over them to create two new potential solutions. Otherwise, plot the new points over the parent points. Finally, you mutate the new points and generate the resulting points.

The way that selection occurs is by comparing potential parents with potential partners in its local area. The values with a higher fitness value are more likely to produce offspring than those with lower fitness to better simulate evolution. Selection is often made by random chance, with the high fitness results being more likely to be picked. The probability of selection (pi) is represented by equation (1), with *f*_*i*_ being the fitness value of individual *i* and *N* being the local population relative to a parent:

(1) $$\begin{array}{c} p_{i}=\frac{f_{i}}{\sum_{j=i}^{N}f_{j}}. \end{array}$$

The algorithm uses a crossover process to generate two new values to plot into the next iteration when selection is complete. These new values perturb old solutions as they try to steer away from the flaws. The general equation for the crossover stage is shown below for *y*_*k*_ and *x*_*k*_, respectively, where *α* is the crossover blending factor and *r*_*k*_ is the uniformly distributed random number in the interval [0, 1]. However, some highly successful members of the next iteration are allowed to remain the same as they were beforehand:

(2) $$\begin{array}{c} y_{k}=\left(1+2\alpha \right)r_{k}-\alpha ,\\ x_{k}^{\left(i,t+1\right)}=\left(1-y_{k}\right)x_{k}^{\left(1,t\right)}+y_{k}x_{k}^{\left(2,t\right)},k=1,\ldots ,n\mathrm{var}. \end{array}$$

To prevent the new iterations from becoming the same and promote more out-of-the-box solutions, a mutation factor is used to diversify the solutions and prevent the population from becoming stagnant. A mutation is a deviation from the crossover logic, which randomizes the solutions generated to hurl them closer or further from the end goal or toward another goal. The equations used to determine the mutation effect is shown below, with *r* being a uniformly distributed number in the interval [0, 1], *x*_*k*_^*l*^ and *x*_*k*_^*u*^ being the upper and lower bounds of *x*_*k*_*T* is the number of generations, *T* is the maximum number of generations, *b* is the strength of the mutation operator, and the function for *y* is given by Δ(*t*, *y*):

(3) $$\begin{array}{c} x_{k}^{\left(i,t+1\right)}=x_{k}^{\left(1,t\right)}+\Delta \left(t,x_{k}^{u}-x_{k}^{\left(2,t\right)}\right)r\leq 0.5,k=1,\ldots ,n,\\ x_{k}^{\left(i,t+1\right)}=x_{k}^{\left(1,t\right)}-\Delta \left(t,x_{k}^{\left(2,t\right)}-x_{k}^{\left(2,t\right)}\right)r>0.5,k=1,\ldots ,n,\\ \Delta \left(t,y\right)=y{\left(1-r^{1-t/T}\right)}^{b}. \end{array}$$

### 2.2. Particle Swarm Optimization

In 1995, electrical engineer Russel Eberhart and social psychologist James Kennedy developed this alternative to the genetic algorithm. This nongradient algorithm considers the individuality and sociability of the population members. Specifically, the idea came from watching birds look for a nesting place. Not enough individuality led to too many birds trying to nest in the same place. However, not enough sociability led to many birds unable to find suitable nesting places. In general, the program uses social rules and individual deviations to find the ideal locations. It is calculated by accounting for the velocity vector of each particle as they travel. The vector considers the pack movement and individual instinct that goes into its movement and adds it to the initial inertia of the iteration. The basic equation for particle swarm vector optimization is shown below, with *α* being the inertia factor, *β*_1_ being the individuality factor, *β*_2_ being the sociability factor, *r*_1_^(*i*)^ and *r*_2_^(*i*)^ being uniformly distributed numbers in the interval [0, 1], *X*^(*i*, *t*)^ being the individual's vector, *P*^(*i*)^ being the best individual value and *P*^(*i*)^ being the best value in the population. Within the vector equation, *αv*^(*i*, *t*)^ represents the inertia, *β*_1_*r*_1_^(*i*)^(*P*^(*i*)^ − *X*^(*i*, *t*)^) represents the individuality, and *β*_2_*r*_2_^(*i*)^(*P*^(*g*)^ − *X*^(*i*, *t*)^) represents sociability:

(4) $$\begin{array}{c} v^{\left(i,t+1\right)}=\alpha v^{\left(i,t\right)}+\beta_{1}r_{1}^{\left(i\right)}\left(P^{\left(i\right)}-X^{\left(i,t\right)}\right)+\beta_{2}r_{2}^{\left(i\right)}\left(P^{\left(g\right)}-X^{\left(i,t\right)}\right),\\ X^{\left(i,t+1\right)}=X^{\left(i,t\right)}+v^{\left(i,t+1\right)}. \end{array}$$

Other than this, it functions like the genetic algorithm; it begins with many solutions on the field. Each solution is evaluated for fitness. The result is compared to their previous swarm fitness, and the previous individual fitness and its position are updated accordingly. Its best individual fitness and position are then used to calculate the next iteration.

All in all, particle swarm optimization edges out over the genetic algorithm, namely, because it does not need to sort fitness as the genetic algorithm does. It means that swarm optimization requires less-computational power. It tends to be cheaper to use than the genetic algorithm, especially with many values.

### 2.3. Differential Evolution

Differential evolution was developed around 1955 and was made to try simulating Darwinian evolution. It combines the parents' features to form a child. However, unlike previous methods, the new value may inherit features from multiple parents. It is the closest to gradient optimization that evolution optimization can get in this assignment. It is used for multidimensional real-valued functions without needing it to be differentiable, making it a robust algorithm.

Using two different parent equation values (*P*1 and *P*2), the method produces a series of children (*C*1,…,*C*_*n*_). In these equations, *α*, *β*, and *γ* are random parent features, *m* is the mutation factor between 0.5 and 1, and *δ*_1_ and *δ*_2_ are binomial crosses over coefficients. CR is the crossover, while *R* represents a random number with distribution [0, 1]:

(5) $$\begin{array}{c} P_{n}=\alpha +m\left(\beta -\gamma \right),\\ C_{n}=P_{2}\delta_{1}+P_{1}\delta_{2},\\ X_{i}^{k+1}=\delta_{1}X_{i}^{k}+\delta_{2}\left(\alpha +m\left(\beta -\gamma \right)\right),\\ \delta_{1}=0\left(\text{if }R<CR\right),\\ \delta_{1}=1\left(\text{if }R>CR\right),\\ \delta_{2}=0\left(\text{if }R>CR\right),\\ \delta_{2}=1\left(\text{if }R<CR\right). \end{array}$$

It is an algorithm that only acts when the product of the two-parent points produces a child with better fitness. When weighing its options on its results, it always selects the offspring with the excellent fitness. It abandons the rest, increasing the efficiency of the evolution. Furthermore, any improvements found by the function will be immediately included. As a result, the general solution is often more accurate than in either the genetic algorithm or particle swarm optimization.

## 3. Results and Discussion

**Figure 1:**
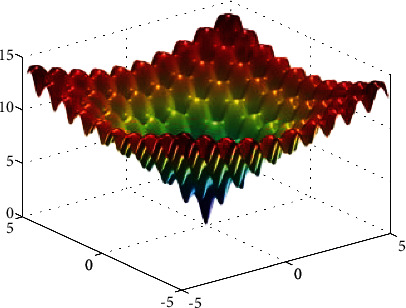
Ackley function.

**Figure 2:**
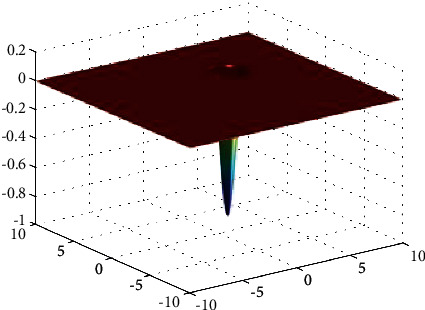
Easom function.

**Figure 3:**
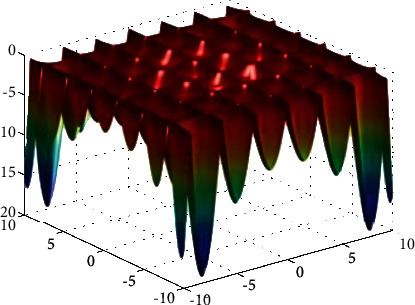
Holder table function.

**Figure 4:**
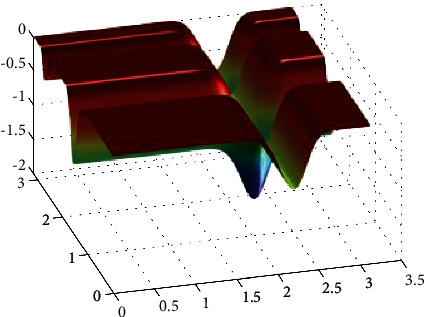
Michalewicz function.

**Figure 5:**
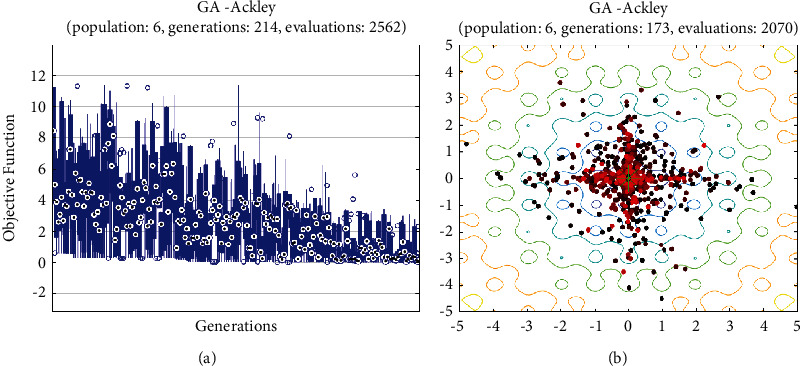
Genetic algorithm results for Ackley function: (a) objective function in each generation, (b) plot of populations accumulation.

**Figure 6:**
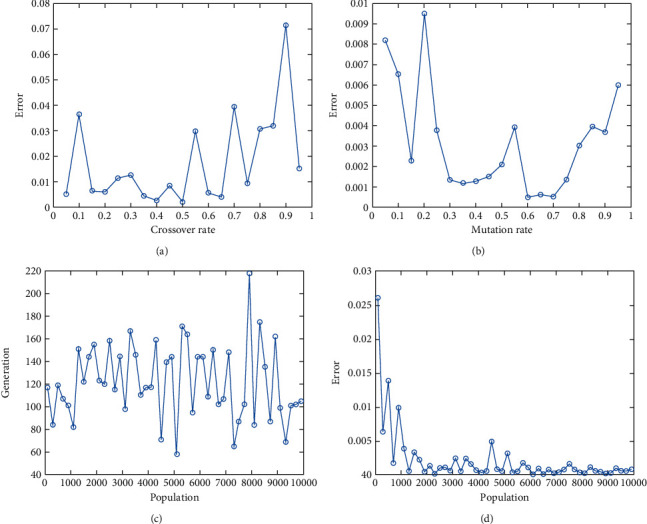
Genetic algorithm results for Ackley function: (a) error value based on increasing on crossover rate, (b) error value based on the mutation rate, (c) number of generation vs. population, (d) Error value with increase in population.

**Figure 7:**
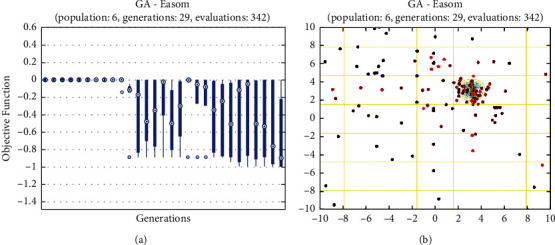
Genetic algorithm results for Easom function: (a) objective function in each generation, (b) plot of populations accumulation.

**Figure 8:**
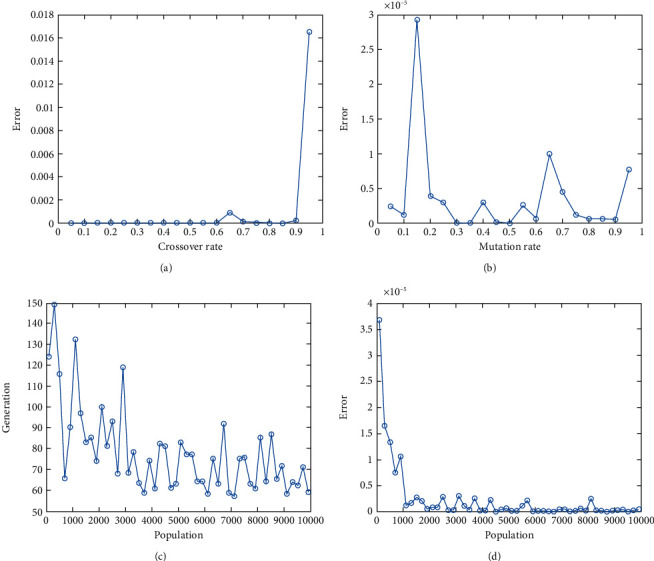
Genetic algorithm results for Easom function: (a) error value based on increasing on crossover rate, (b) error value based on the mutation rate, (c) number of generation vs. population, (d) error value with increase in population.

**Figure 9:**
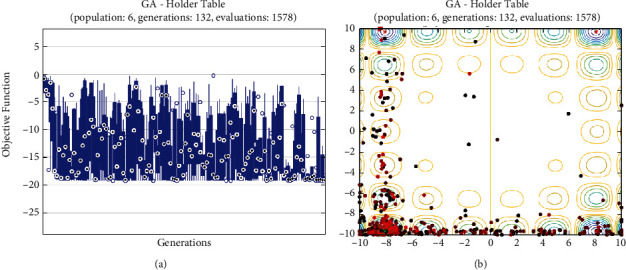
Genetic algorithm results for holder table function: (a) objective function in each generation, (b) plot of populations accumulation.

**Figure 10:**
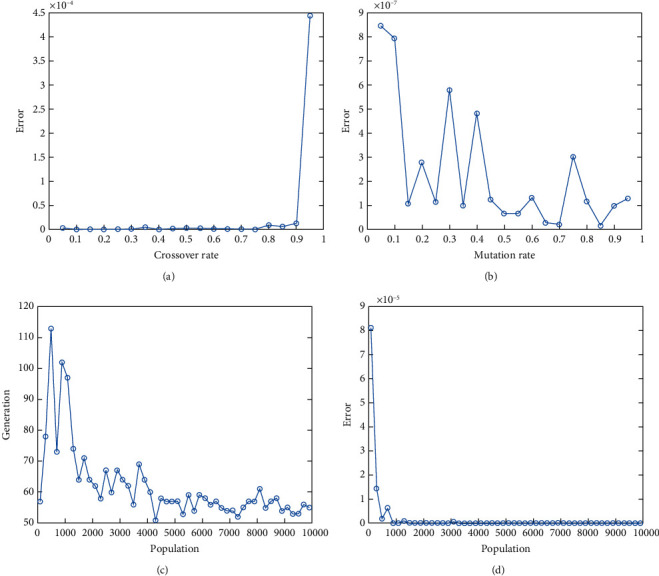
Genetic algorithm results for holder table function: (a) error value based on increasing on crossover rate, (b) error value based on the mutation rate, (c) number of generation vs. population, (d) error value with increase in population.

**Figure 11:**
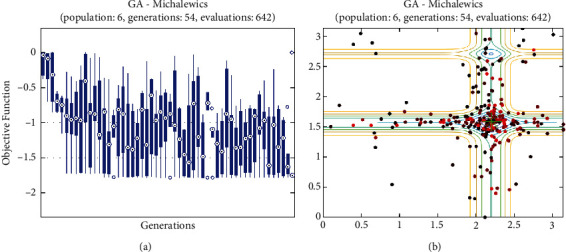
Genetic algorithm results for Michalewicz function: (a) objective function in each generation, (b) plot of population accumulation.

**Figure 12:**
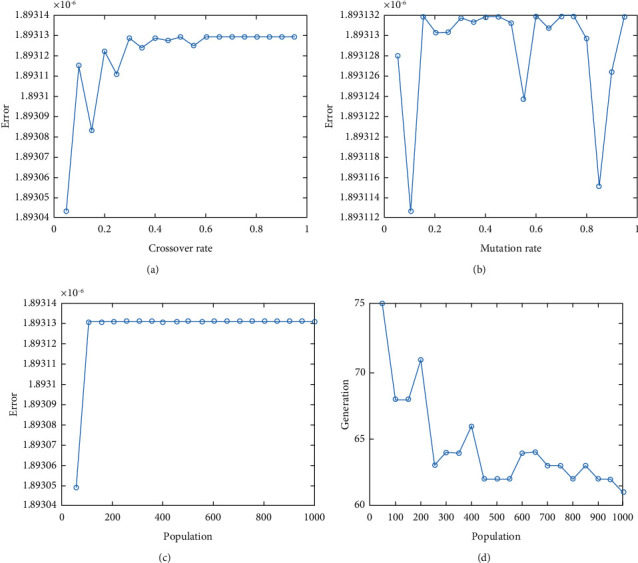
Genetic algorithm results for Michalewicz function: (a) error value based on increasing on crossover rate, (b) error value based on the mutation rate, (c) number of generation vs. population, (d) error value with increase in population.

In this report, we used three meta-heuristic algorithms of genetic algorithm, particle swarm optimization, and differential evaluation as two nongradient-based methods for optimization of some mathematical surfaces. In this report, 12 functions of Easom, Holder table, Michalewicz, Ackley, Rastrigin, Rosen, Rosenbrock, Shubert, Sphere, Schaffer, Himmelblau's, and Spring Force Vanderplaats are employed for the optimization. The properties of these functions are as follows (see Table 2):

In this report, we used GA to optimize Easom, Holder table, Michalewicz, Ackley functions shown in Figures 1–12. Moreover, Figures 5, 7, 9, and 11 illustrate the objective function values in each generation of genetic algorithm and plot of populations accumulation to find the optimum value. Furthermore, Figures 6, 8, 10, and 12 show the genetic algorithm error value based on increasing on crossover rate mutation rate. In the number of generations versus population, error values increase with increase in population. Based on the analysis results, the best value of crossover rate for optimization of Ackley function is 0.4–0.5, and mutation rate is 0.6–0.7 (Table 3).

Moreover, based on Figure 6(c), it can be estimated that with the increase of the population to 10,000, there is no significant increase/decrease in the number of generations in Ackley function. Therefore, GA can find the optimum value with the minimum population value. The GA method is low complexity in finding the global minimum of the Ackley function. Furthermore, based on Figure 6(d), the minimum population value for reaching the best complexity is 1000. Easom and Holder table functions results are shown in Figures 7–10. Based on the results, there are no significant effects between changing crossover, mutation rate, and error value because with the small population and 100 generations, GA can find the minimum value of the function. Regarding the results of Michalewicz function with the increase of the number of populations, generation is decremented. However, there is no optimum value of crossover mutation rate for this function because of less complexity of GA for optimization of these functions.

**Figure 13:**
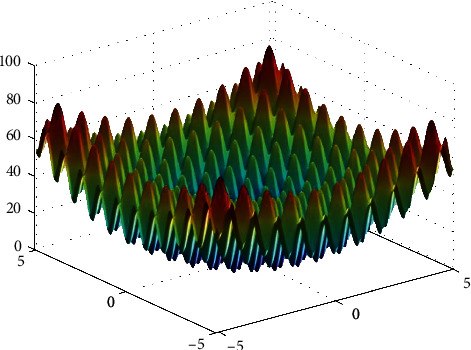
Rastrigin function.

**Figure 14:**
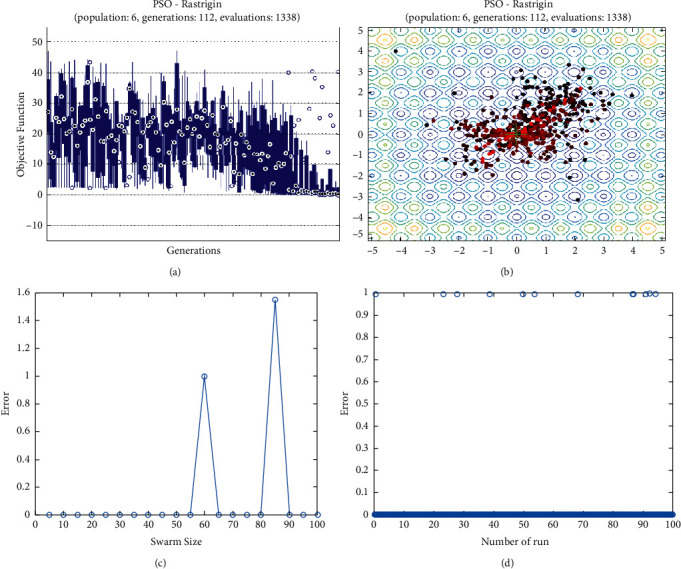
PSO results for Rastrigin function: (a) objective function in each generation, (b) plot of populations accumulation, (c) error value with variation f swarm size, (d) error value with repetition of a single run.

**Figure 15:**
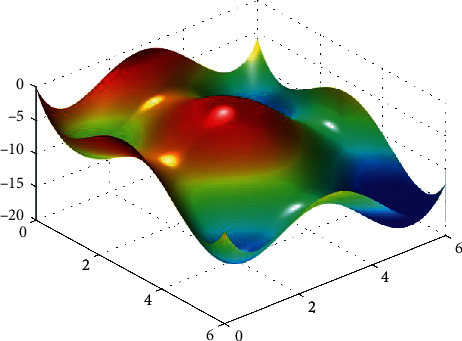
Rosen function.

**Figure 16:**
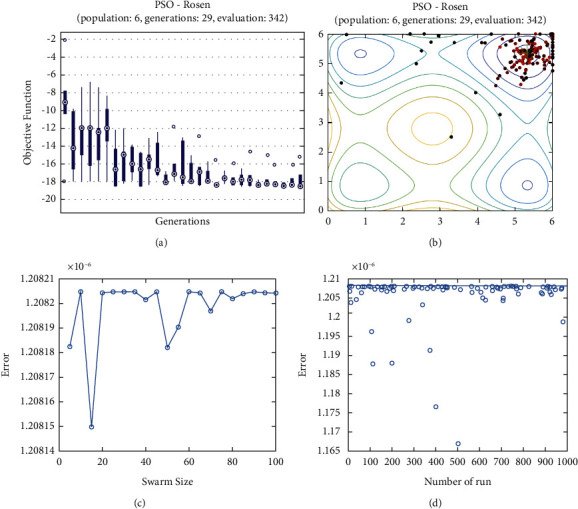
PSO results for Rosen function: (a) objective function in each generation, (b) plot of populations accumulation, (c) error value with variation f swarm size, (d) error value with repetition of a single run.

**Figure 17:**
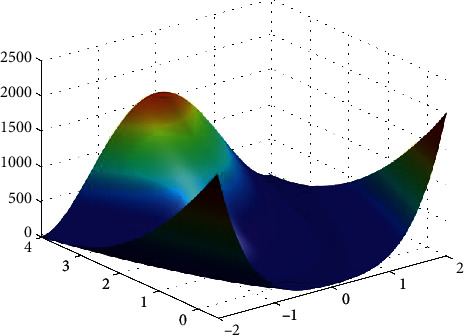
Rosenbrock function.

**Figure 18:**
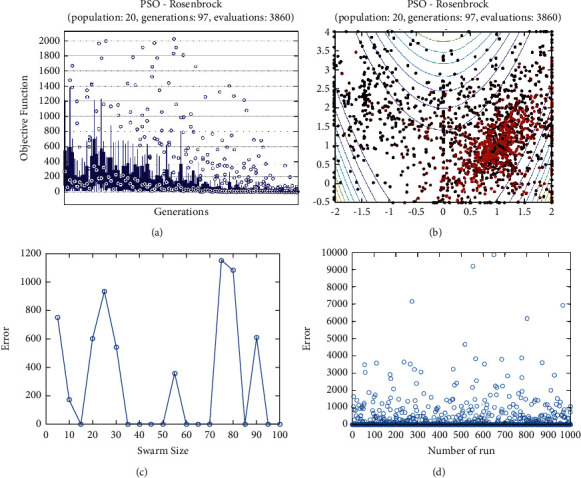
PSO results for Rosenbrock function: (a) objective function in each generation, (b) plot of populations accumulation, (c) error value with variation f swarm size, (d) error value with repetition of a single run.

**Figure 19:**
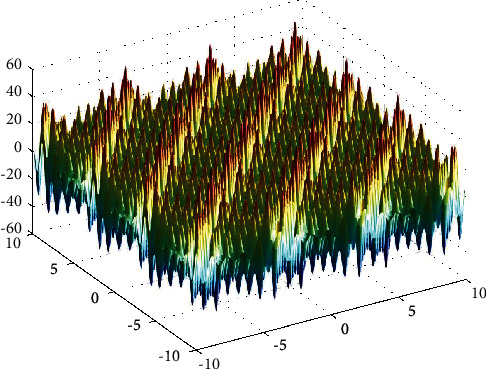
Shubert function.

**Figure 20:**
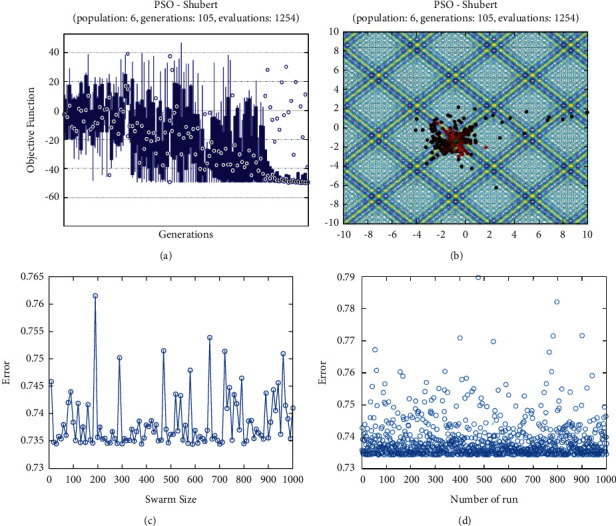
PSO results for Shubert function: (a) objective function in each generation, (b) plot of populations accumulation, (c) error value with variation *f* swarm size, (d) error value with repetition of a single run.

For testing the PSO, the effects of swarm size are compared for each of Rastrigin, Rosen, Rosen Brock, Shubert functions (Figures 13–17. Based on the results, two of 60 and 85 swarms have not accurate results. Therefore, we repeat the optimization 1000 times with a specific swarm size. It can be seen that 1% of evaluations cannot find the optimum value of Rastrigin function (seen Figure 18(d)). However, for the Rosen function, 100% of runs are accurate. One of the complicated formulas in optimization is the Rosenbrock function, based on the results, many runs are not accurate results regarding Figure 19(d). Moreover, there is no relationship between swarm size and optimization accuracy, because sometimes PSO cannot find the optimum value. These results are also repeated in the Shubert function in Figure 20 based on the results, PSO does not have higher robustness for finding the optimum value of these function types because it can no longer be reliable results at least these equations.

**Figure 21:**
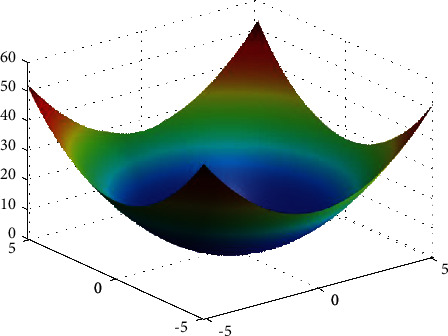
Sphere function.

**Figure 22:**
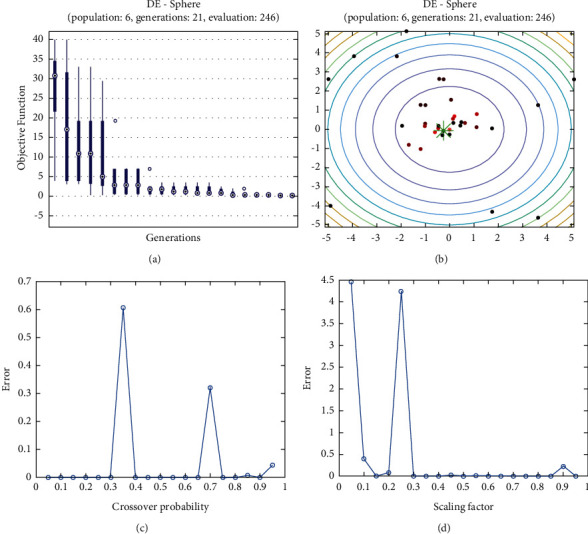
DE results for sphere function: (a) objective function in each generation, (b) plot of population accumulation, (c) error value with the variation of crossover probability, (d) error value changing of scaling function.

**Figure 23:**
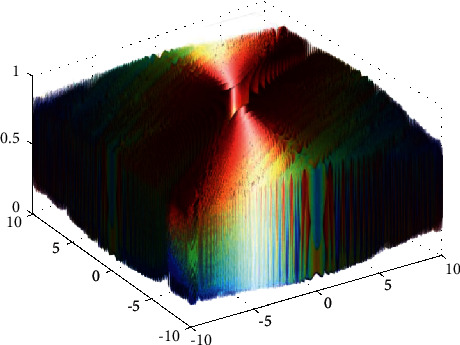
Schaffer function.

**Figure 24:**
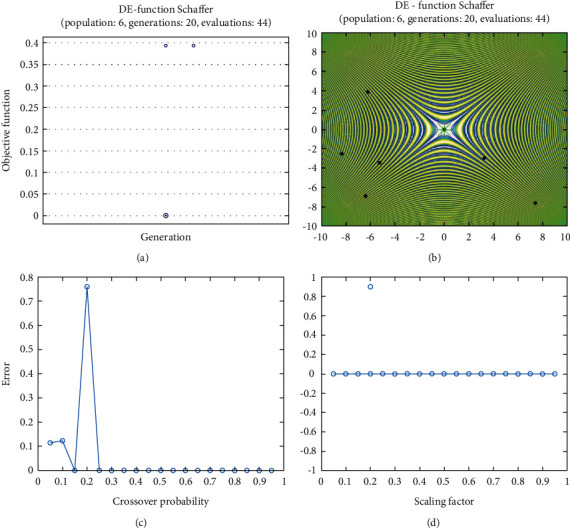
DE results for Schaffer function: (a) objective function in each generation, (b) plot of population accumulation, (c) error value with the variation of crossover probability, (d) error value changing of scaling function.

**Figure 25:**
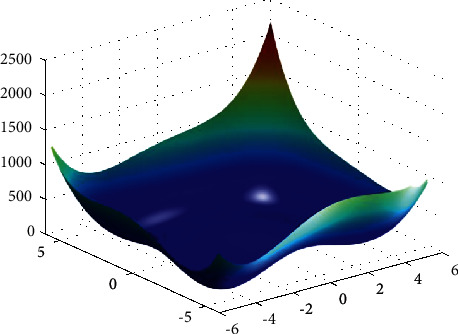
Himmelblau function.

**Figure 26:**
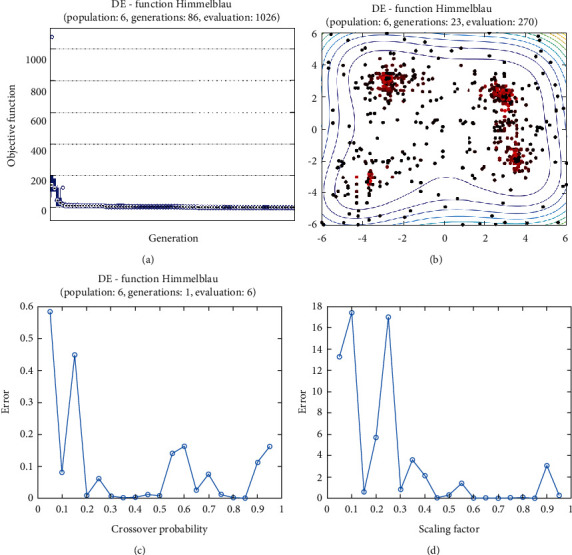
DE results for Himmelblau function: (a) objective function in each generation, (b) plot of population accumulation, (c) error value with the variation of crossover probability, (d) error value changing of scaling function.

**Figure 27:**
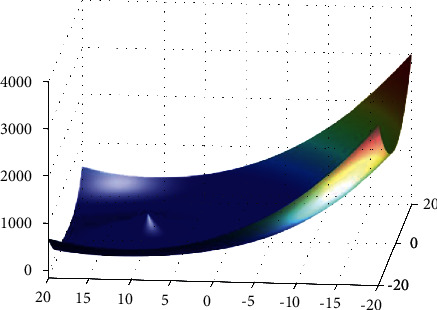
Spring force Vanderplaats function.

**Figure 28:**
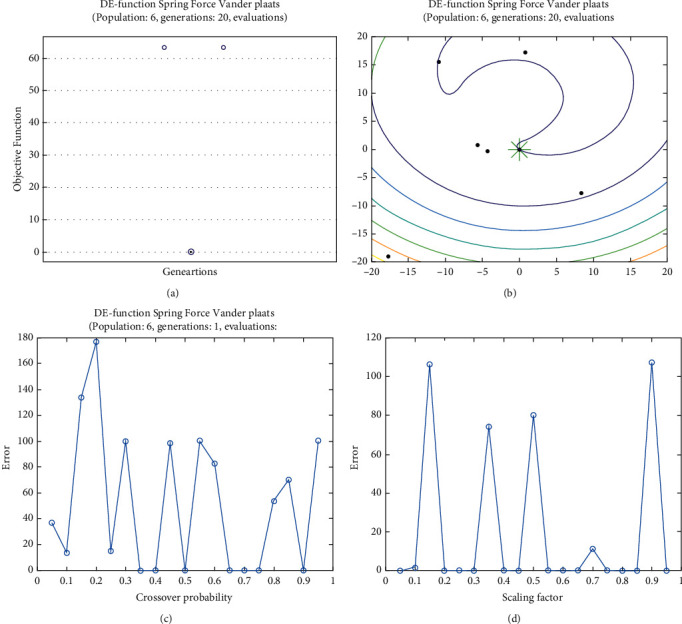
DE results for spring force Vanderplaats function: (a) objective function in each generation, (b) plot of populations accumulation, (c) error value with the variation of crossover probability, (d) error value changing of the scaling function.

**Figure 29:**
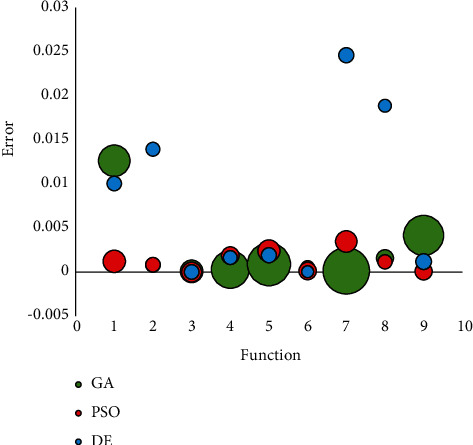
The comparison of GA, PSO, and DE.

For analysis of DE algorithms, four Sphere, Schaffer, Himmelblau's, and Spring Force Vanderplaats are used. Figures 21–24 depict the 3D surface of the following equations, and Figures 25–28 illustrate the DE evaluation results. We tested the crossover rate and scaling factor in the accuracy of the DE method. Based on the results for optimization Sphere, the best scaling factor is 0.3. There is no relationship between error and crossover rate for crossover rate. Overly, one of the properties of DE is using a lower number of initial populations with lower time complexity to find the optimum value of the functions. However, it is sensitive in choosing the crossover rate. Based on Figure 27, the optimum crossover value is 0.3, and the scaling factor is 0.45. Moreover, in DE, there is no relationship between the crossover and scaling factor rate on error for the spring force Vanderplaats function (see Figure 29).

In the next step all the nine (1) Ackley, (2) Easom, (3) Holder table, (4) Michalewicz, (5) Rastrigin, (6) Rosen, (7) Rosenbrock, (8) Sphere, and (9) Himmelblau's are tested using GA, PSO, and DE algorithm. For all the functions, number of the population is identical and 20 (see Table 4).

Based on the comparison results between the optimization methods, the DE algorithm has the lowest time complexity among other methods. Moreover, GA illustrated the highest time complexity. However, the PSO algorithm has lower reliability to find the optimum point.

## 4. Conclusion and Future Works

The objective of this report is to evaluate nongradient-based methods for optimizing some mathematical surfaces by applying three meta-heuristic algorithms, including genetic algorithms, particle swarm algorithms, and differential evaluation algorithms. In this report, 12 functions of Easom, Holder table, Michalewicz, Ackley, Rastrigin, Rosen, Rosen Brock, Shubert, Sphere, Schaffer, Himmelblau's, and Spring Force Vanderplaats are used for optimization. We utilized GA to optimize Easom, Holder tables, Michalewicz, and Ackley functions in this report. The number of generations versus the population, error value as the population increases. According to the results of the analysis, the best crossover rate for optimization of the Ackley function is 0.4–0.5, and the best mutation rate is 0.6–0.7. For GA, it is estimated that with the increase in population to 10,000, there is no significant increase or decrease in the number of generations in Ackley function. Consequently, GA is able to find the optimal value with a minimum population value. Using the GA method, the global minimum of the Ackley function can be determined with a low degree of complexity. Additionally, the minimum population value for the best degree of complexity is 1000. There are no significant effects of changing crossover, mutation rate, and error value for Easom and Holder table functions. Michaelewicz function shows that generation decreases with an increase in the number of populations. Due to the simplicity of GA in optimizing these functions, there is no optimal crossover mutation rate for this function.

In order to test the PSO, the effects of swarm size are compared for Rastrigin, Rosen, Rosenbrock, and Shubert functions. The optimization is repeated 1000 times with the same swarm sizes. It can be seen that 1% of evaluations are not able to determine the optimum value for the Rastrigin function. In contrast, 100% of evaluations are able to determine the Rosenbrock function. The Rosenbrock function is one of the most complex formulas in optimization. According to the results, there is no relationship between swarm size and optimization accuracy. These results indicate that PSO does not have higher robustness for finding optimum values of these function types since it is no longer able to produce reliable results, at least for these equations. An analysis of DE algorithms uses four Spheres, Schaffers, Himmelblaus, and Spring Force Vanderplaats. To test the accuracy of the DE method, we tested the crossover rate and scaling factor. According to the results for optimization Sphere, the best scaling factor is 0.30. In terms of the crossover rate, there is no relationship between error and crossover rate. In general, one of the characteristics of DE is that it uses fewer initial populations with a shorter time complexity to find the optimal values. It is sensitive to the crossover rate, however. Furthermore, there is no relationship between the crossover and the scaling factor rate on error for the spring force Vanderplaats function in DE. Comparing the results of the optimization methods, it appears that the DE algorithm has the lowest time complexity. The GA algorithm has the highest time complexity. In contrast, the PSO algorithm is less reliable for finding the optimum point.

The use of meta-heuristics has enabled engineers to solve several engineering problems that could not be solved with standard optimization approaches. Examples include the simplicity with which they can be combined in finite element software in any domain, where the combination/permutation of solutions available to each method enables the discovery of optimum projects without the need of explicit functions. Literature contains numerous examples of this phenomenon. Developing a meta-heuristic that can accomplish this with fewer populations and iterations (lower processing costs) and more accuracy is the point of contention in the literature between new algorithms attempting this goal. If the algorithm is evolutionary in nature, swarms, behaviors, and physical occurrences are all features that contribute to the primary purpose outlined above. I believe that the universal law of time will reveal those algorithms that are truly superior and distinguishable from the others. Additionally, as a reviewer, you may request tests such as Wilcoxon to determine whether the way each meta-heuristic operates has changed.

## Figures and Tables

**Table 1:** Review of nongradient-based methods and their application.

Author	Year	Gradient-based method	Application	Results
Chen et al. [21]	2022	Evolutionary optimization	Learning variational quantum reinforcement	It provided a natural way to compress the input dimension efficiently, enabling further quantum RL uses on noisy intermediate-scale quantum devices

Zhang et al. [2]	2021	Nongradient topology optimization in acoustic meta-materials	Realizing a complete and directional bandgap design	The optimized designs converged to show the orderly material distribution and numerical validations to show expected propagation properties

Pazouki [22]	2021	Volume balance model and multiobjective evolutionary optimization algorithms.	Designing a practical surface irrigation system	The proposed model in most fields and indicators achieve better results, and the results are close together

Dhiman et al. [23]	2021	A new evolutionary multiobjective optimization algorithm for global optimization	To map out seagulls better than modern optimization algorithms	The empirical research indicates that the EMoSOA algorithm works better than other algorithms

Pan et al. [24]	2021	An efficient surrogate-assisted hybrid optimization algorithm	Solving expensive optimization problems	The hybrid algorithm works better than preexisting ones, able to solve problems that were previously unattainable

Abualigah et al. [25]	2021	A novel evolutionary arithmetic optimization algorithm	Multilevel thresholding segmentation of covid-19 ct images	The DAOA produces higher quality solutions than other similar approaches and is ranked the best for various test cases

Naeimi et al. [26]	2021	A nature-inspired algorithm for high-dimensional optimization problems	To develop an algorithm based on horses' herding behaviors for high-dimensional optimization	The proposed algorithm proved to be highly efficient for solving serious dimensional global optimization problems, outperforming the standard algorithms used today in terms of accuracy and efficiency

Meraihi et al. [27]	2021	Genetic algorithm optimization	To develop an algorithm based on the foraging and swarming behaviors of grasshoppers	The GOA algorithm gives superior results for most applications, having a high exploitation ability and convergence and excelling at preventing local minima stagnation.

**Table 2:** The properties of optimization functions.

Function	Variable	*X* domain	*Y* domain	Global minimum	Optimum value
Ackley	2	−5 ≤ *x* ≤ 5	−5 ≤ *y* ≤ 5	*x* ^*∗*^ = (0, 0)	*f*(*x*^*∗*^) = 0
Easom	2	−10 ≤ *x* ≤ 10	−10 ≤ *y* ≤ 10	*x* ^*∗*^ = (*π*, *π*)	*f*(*x*^*∗*^) = −1

Holder table	2	−10 ≤ *x* ≤ 10	−10 ≤ *y* ≤ 10	*x* ^*∗*^ = (8.06, 9.66) *x*^*∗*^ = (−8.06, 9.66) *x*^*∗*^ = (8.06, −9.66) *x*^*∗*^ = (−8.06, −9.66)	*f*(*x*^*∗*^) = −19.21

Michalewicz	2	0 ≤ *x* ≤ *π*	0 ≤ *y* ≤ *π*	*x* ^*∗*^ = (2.20, 1.57)	*f*(*x*^*∗*^) = −1.80
Rastrigin	2	−5 ≤ *x* ≤ 5	−5 ≤ *y* ≤ 5	*x* ^*∗*^ = (0, 0)	*f*(*x*^*∗*^) = 0
Rosen	2	0 ≤ *x* ≤ 6	0 ≤ *y* ≤ 6	*x* ^*∗*^ = (5.33, 5.33)	*f*(*x*^*∗*^) = −18.57
Rosenbrock	2	−2 ≤ *x* ≤ 2	−5 ≤ *y* ≤ 4	*x* ^*∗*^ = (1, 1)	*f*(*x*^*∗*^) = 0
Shubert	2	−10 ≤ *x* ≤ 10	−10 ≤ *y* ≤ 10	*x* ^*∗*^ = (1.67, −2.01)	*f*(*x*^*∗*^) = −186.73
Sphere	2	−5 ≤ *x* ≤ 5	−5 ≤ *y* ≤ 5	*x* ^*∗*^ = (0, 0)	*f*(*x*^*∗*^) = 0
Schaffer	2	−10 ≤ *x* ≤ 10	−10 ≤ *y* ≤ 10	*x* ^*∗*^ = (0, 0)	*f*(*x*^*∗*^) = 0
Himmelblau	2	−6 ≤ *x* ≤ 6	−6 ≤ *y* ≤ 6	*x* ^*∗*^ = (3, 2)	*f*(*x*^*∗*^) = 0
Spring force Vanderplaats	2	−20 ≤ *x* ≤ 20	−20 ≤ *y* ≤ 20	*x* ^*∗*^ = (0, 0)	*f*(*x*^*∗*^) = 0

**Table 3:** The expressions of optimization problems.

Function	Equations
Ackley	$f\left(x,y\right)=-20e^{-0.02\sqrt{x^{2}+y^{2}/2}}-e^{\cos\left(2\pi x\right)+\cos\left(2\pi y\right)/2}+e+20$
Easom	*f*(*x*, *y*)=−cos*x*.cos*y*.exp(−(*x* − *π*)^2^ − (*y* − *π*)^2^)
Holder table	$f\left(x,y\right)=-\left|\sin x.\cos y.\exp\left(\left|1-\sqrt{x^{2}}+y^{2}/\pi \right|\right)\right|$
Michalewicz	*f*(*x*, *y*)=−sin*x*.sin^20^(*x*^2^/*π*) − sin*y*.sin^20^(2*y*^2^/*π*)
Rastrigin	*f*(*x*, *y*)=20+*x*^2^ − 10cos(2*πx*)+*y*^2^ − 10cos(2*πx*)
Rosen	*f*(*x*, *y*)=0.25*x*^4^ − 3*x*^3^+11*x*^2^ − 13*x*+0.25*y*^4^ − 3*y*^3^+11*y*^2^ − 13*y*
Rosenbrock	*f*(*x*, *y*)=(1 − *x*)^2^+100(*y* − *x*^2^)^2^
Shubert	*f*(*x*, *y*)=(∑_*i*=1_^5^(*i*cos(*i*+1)*x*+*i*))(∑_*i*=1_^5^(*i*cos(*i*+1)*x*+*i*))
Sphere	*f*(*x*, *y*)=*x*^2^+*y*^2^
Schaffer	*f*(*x*, *y*)=1/2+sin^2^(*x*^2^+*y*^2^)+0.5/(1+0.001(*x*^2^+*y*^2^))^2^
Himmelblau	*f*(*x*, *y*)=*f*=(*x*^2^+*y* − 11)^2^+(*x*+*y*^2^ − 7)^2^
Spring force Vanderplaats	$f\left(x,y\right)=4{\left(\sqrt{x^{2}+{\left(10-y\right)}^{2}}-10\right)}^{2}+1/2{\left(\sqrt{x^{2}+{\left(10-y\right)}^{2}}-10\right)}^{2}-5x-5y$

**Table 4:** The comparison of GA, PSO, and DE using error and runtime metrics.

No	Function	GA		PSO		DE	
		Error	Runtime	Error	Runtime	Error	Runtime
1	Ackley	0.012625	21.00289	0.001218	10.63231	0.010042	4.467835
2	Easom	0.000807	4.485303	0.000836	4.488985	0.013936	4.121538
3	Holder table	0.000082	10.84023	0	8.384456	0.000012	4.529235
4	Michalewicz	0.000303	30.27151	0.00188	6.489204	0.001643	4.065657
5	Rastrigin	0.000894	39.14045	0.002408	10.31332	0.001909	4.802492
6	Rosen	0.000541	3.833337	0.000124	6.523122	0.000042	3.013651
7	Rosenbrock	0.00014	45.31977	0.003471	9.796138	0.024601	4.906589
8	Sphere	0.001576	6.141895	0.001154	4.310654	0.018867	3.522635
9	Himmelblau's	0.004158	33.9344	0.000077	6.249301	0.00119	5.255951

## Data Availability

Data are available and can be provided over the e-mails querying directly to the author at the corresponding author (amin.valizadeh@mail.um.ac.ir).

## Nomenclature

*p*_*i*_: Probability of selection
*f*: Objective function
*N*: Number of populations
*α*: Crossover blending factor
*r*_*k*_: Random number
*t*: Generation
*β*_1_: Individuality factor
*β*_2_: Sociability factor
*α*: PSO inertia factor
*δ*1 and *δ*2: Random parent features.
